# Screening and identification of a six-cytokine biosignature for detecting TB infection and discriminating active from latent TB

**DOI:** 10.1186/s12967-018-1572-x

**Published:** 2018-07-20

**Authors:** Sen Wang, Yang Li, Yaojie Shen, Jing Wu, Yan Gao, Shu Zhang, Lingyun Shao, Jialin Jin, Ying Zhang, Wenhong Zhang

**Affiliations:** 1 Department of Infectious Diseases, Institute of Infectious Diseases, Huashan Hospital, Fudan University, 12 Wulumuqi Zhong Road, Shanghai, 200040 China; 2 Institutes of Biomedical Sciences, Fudan University, Shanghai, 200032 China; 3 MOH and MOE Key Laboratory of Medical Molecular Virology, Shanghai Medical College, Fudan University, Shanghai, 200032 China; 4 Department of Molecular Microbiology and Immunology, Bloomberg School of Public Health, Johns Hopkins University, Baltimore, MD 21205 USA

**Keywords:** TB, Multiplex, Diagnostic, Cytokines

## Abstract

**Background:**

The early and accurate diagnosis of tuberculosis (TB) is critical for controlling the global TB epidemic. Although early studies have supported the potential role of cytokine biomarkers in blood for the diagnosis of TB, this method requires further investigation and validation in different populations. A set of biomarkers that can discriminate between active TB (ATB) and latent TB infection (LTBI) remains elusive.

**Methods:**

In the current study, we organized two retrospective cohorts and one prospective cohort to investigate the immune responses at different clinical stages of TB infection, as determined by candidate cytokine biomarkers detected with a multiplex cytokine platform. Using a pre-established diagnostic algorithm, participants were classified as ATB, LTBI, and TB uninfected controls (CON). Based on our multiplex cytokine assay, a multi-cytokine biosignature was modelled for the optimal recognition of the different TB infection status.

**Results:**

Our analysis identified a six-cytokine biosignature of TB-antigen stimulated IFN-γ, IP-10, and IL-1Ra, and unstimulated IP-10, VEGF, and IL-12 (p70) for a biomarker screening group (n = 88). The diagnostic performance of the biosignature was then validated using a biomarker validation cohort (n = 216) and resulted in a sensitivity of 88.2% and a specificity of 92.1%. In a prospectively recruited clinical validation cohort (n = 194), the six-cytokine biosignature was further evaluated, and displayed a sensitivity of 85.7%, a specificity of 91.3% and an overall accuracy of 88.7%.

**Conclusions:**

We have identified a six-cytokine biosignature for accurately differentiating ATB patients from subjects with LTBI and CON. This approach holds promise as an early and rapid diagnostic test for ATB.

**Electronic supplementary material:**

The online version of this article (10.1186/s12967-018-1572-x) contains supplementary material, which is available to authorized users.

## Background

Tuberculosis (TB) remains a leading cause of morbidity and mortality worldwide. In 2015, there were 10.4 million annual cases [1]. It has been estimated that a third of the world’s population is infected with *Mycobacterium tuberculosis* (*Mtb*), and each year 1.4 million people die from TB. The majority of *Mtb* infections remain asymptomatic, establishing latent TB infections (LTBI), but up to 10% can progress into active TB, becoming contagious during a period of months to decades after the initial infection [2]. Thus, the early detection and initiation of treatment for active TB (ATB) as well as LTBI are essential for intensifying the fight against TB and implementing the End TB Strategy [3, 4].

Based on the immunological sensitization to specific mycobacterial antigens, interferon-γ (IFN-γ) release assays (IGRAs) were developed for the diagnosis of *Mtb* infection. There are two IGRAs commercially available today, including the whole-blood aided enzyme-linked immunosorbent assay (ELISA)-based QuantiFERON-Gold in tube (QFT) test and the peripheral blood mononuclear cell (PBMC) aided enzyme-linked Immunospot assay-based T-SPOT.TB test. Both IGRAs incorporate the region of difference 1 (RD1)-encoded 6 kDa early secretory antigenic target (ESAT-6) and 10 kDa culture filtrate protein (CFP-10) antigens, whereas an additional single peptide from the RD11 encoded TB7.7 (Rv2654) is included in the QFT test [5, 6]. Current IGRAs based on the strength of *Mtb*-specific antigens perform better than the traditional tuberculin skin test in their ability to discriminate *Mtb* infections from Bacillus Calmette–Guérin (BCG) vaccination or non-tuberculous mycobacterial infections [7, 8]. However one major limitation of IGRAs is that they fail to distinguish between ATB and LTBI, and this greatly hampers the early treatment and control of TB [9]. The research and development of effective diagnostic tests for TB and the accurate identification of biomarkers for different disease statuses are therefore urgently required.

One alternative solution to improve and support the current immune-based diagnostic tests for ATB would be the identification of alternative cytokine biomarkers to IFN-γ [10]. In addition, the lack of sensitivity and specificity of current IGRAs tests also illustrate that a single biomarker is unlikely to fulfill the requirements for a reliable discrimination between ATB and other pulmonary or related infections. As a consequence, the search for a combination of biomarkers (of so-called biosignatures) to tackle this issue is ongoing. Currently, a number of new cytokine biomarkers, such as EGF, sCD40L, and VEGF [11] or TNF-α, IL-12 p40, and IL-17 [12] are credited to be promising candidates for the immunodiagnostic of active TB or LTBI.

However, current studies involving biomarker screening have a number of limitations. Firstly, most of these studies focus on identifying biomarkers for discriminating between ATB patients and healthy controls, while only a few paid attention to cytokine biomarkers for differentiating between ATB and LTBI. To our knowledge, no large confirmatory biomarker studies for discriminating between ATB and LTBI in the peripheral blood have been carried out [13]. Secondly, several studies detect cytokines that are released directly from the peripheral blood or serum of the test subjects [14–18]. Although these represent a relatively simple examination approach, unstimulated cytokine profiles may be affected by many factors, including inflammation caused by pathogens other than *Mtb*. Finally, for various candidates [19], most of the data from these studies are preliminary and need further validation in ongoing multi-cohort studies, especially in a clinically oriented setting in highly TB-endemic regions, which include subjects with different clinical conditions.

In the light of these limitations, this study aimed to identify *Mtb*-specific cytokine biomarkers that characterize the different status of *Mtb* infections including ATB and LTBI, to determine their diagnostic performance and operational characteristics. Using a prospective clinical cohort, we confirmed that multiple cytokine biomarkers achieved high diagnostic performance in discriminating between the different statuses of TB infections. Moreover, we believe that the application of these new biomarkers may increase the diagnostic yield of the currently available cytokine-based tests.

## Methods

### Study design and case definitions

#### Biomarker screening and validation group

**Fig. 1:**
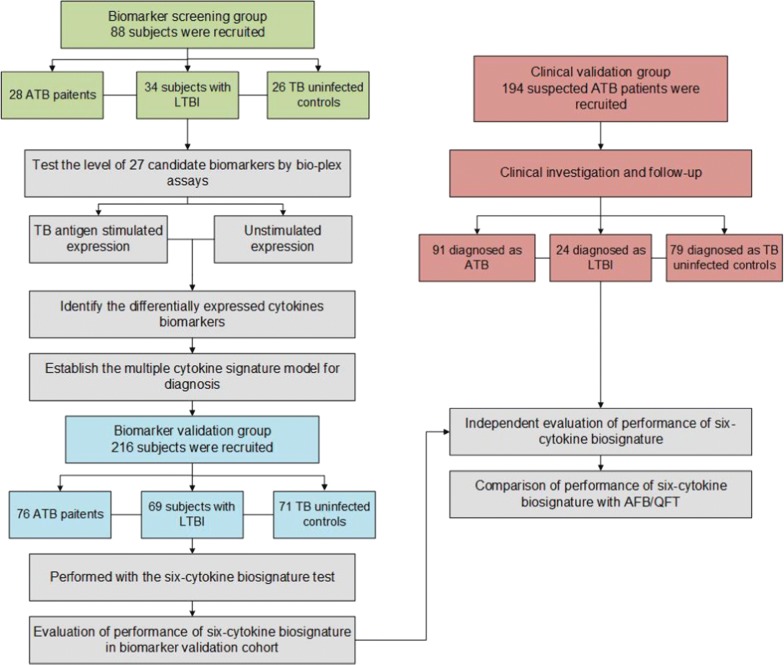
Overall study design and subjects in the biomarker screening, biomarker validation and clinical validation cohorts

To explore and further validate a reliable and effective cytokine biosignature for the diagnosis of TB, we successively organized three groups including a biomarker screening group, a biomarker validation group, and a clinical validation group. The overall study design and classification of study participants were shown in Fig. 1. The biomarker screening group, aiming to identify candidate cytokines associated with the different status of TB infections, enrolled a total of 88 individuals, including 28 patients with ATB, 34 subjects with LTBI, and 26 TB uninfected controls (CON). Based on the results from this group, we constructed a multiple cytokine signature model, as the classifier with the best diagnostic performance using a support vector machine (SVM) analysis. We then assessed the performance of the selected cytokine biosignature in an independent biomarker validation group including 76 ATB, 69 LTBI, and 71 CON subjects.

For the biomarker screening and biomarker validation groups, all the participants were recruited from Shanghai Huashan Hospital, Chongqing Pulmonary Hospital and Zhuji People’s Hospital. Patients with ATB were defined as participants with a clinical condition consistent with ATB such as cough, fever, sputum, lymphadenopathy, chest infiltrations and microbiological findings with evidence of at least two specimens that could confirm the presence of acid fast bacilli (AFB) or at least one specimen can be confirmed in culture as an *Mtb* complex. To minimize the effects of the anti-TB treatment on T cell responses, only untreated patients or those receiving standard anti-TB therapy for < 1 week were included in the study. The LTBI subjects were all recruited from household contacts of patients with ATB. QFT tests and routine radiographic evaluations were performed on all household contacts and subjects who displayed a positive QFT test, and negative chest radiography; no clinical symptoms or evidence of ATB were defined as LTBI. Subjects in the CON group were recruited from subjects who came to the same hospital for an unrelated health examination. For these control subjects, we conducted a questionnaire survey to exclude factors that may affect the outcome such as clinical or radiographic evidence of ATB, known history of exposure to ATB patients, other inflammatory process, and so on. QFT tests were also performed on all subjects and only those with negative QFT results, no clinical or radiographic evidence of active TB, and no known history of exposure to TB were enrolled.

#### Clinical validation group

Following biomarker validation, we further externally examined the clinical application of these biomarkers in a clinical setting-oriented group from a prospective study (clinical validation group) at Shanghai Huashan hospital. Serum samples from 194 ATB suspects were obtained and analyzed using the bio-plex assay. The ATB suspects were defined as patients who presented clinical symptoms (night sweats, weight loss, or cough) or radiographic characteristics consistent with ATB. After a follow-up of at least 3 months, ATB was finally diagnosed using the same criteria as described above and the patients who did not meet the criteria were identified as subjects without ATB disease (NTB). Using prospectively collected clinical data on these subjects, the diagnostic performance of the cytokine signatures was assessed and compared with that of other commonly used clinical diagnostic methods.

All the subjects in our study were consecutively recruited from Shanghai Huashan Hospital Affiliated to Fudan University, Chongqing Pulmonary Hospital, and Zhuji People’s Hospital between August 11, 2009 and January 21, 2016. The study was approved by the Institutional Review Board of Huashan Hospital and the methods were carried out in accordance with the approved guidelines of the institution. Written informed consent was obtained from all participants.

### QFT tests

QFT tests were performed according to the manufacturer’s instructions (Cellestis, Darmstadt, Germany). Briefly, 1 mL of whole blood was drawn into three QFT tubes and incubated at 37 °C within 4 h after collection. Following a 24-h incubation period, the tubes were centrifuged and the plasma was harvested from each tube to determine the concentration of IFN-γ. The QFT results were calculated and interpreted using the manufacturer’s QFT software [20].

### Whole blood assays

Peripheral whole blood samples were collected in heparinized tubes for all the subjects in the biomarker screening, biomarker validation and clinical validation groups. Whole blood samples from the subjects were incubated with the *Mtb* antigens ESAT-6, CFP-10 (each at a concentration of 10 µg/mL; Sangon Peptide Technologies, Shanghai, China), 6-phosphonohexanoic acid (5 µg/mL; positive control; Sigma-Aldrich, St. Louis, MO), or without stimulants (negative controls). Following incubation at 37 °C for 20–24 h, supernatants were harvested and cryopreserved at − 80 °C for batched analysis.

### Quantification of cytokines using the bio-plex assay

To detect and quantify secreted cytokines in cell-free culture supernatants stimulated with ESAT-6 and CFP-10, stimulated or unstimulated whole blood samples were analyzed using the Bio-Plex Pro Human Cytokine kits (Bio-Rad Laboratories, Hercules, CA). Twenty-seven cytokines (IL-1β, IL-1Ra, IL-2, IL-4, IL-5, IL-6, IL-7, IL-8, IL-9, IL-10, IL-12 (p70), IL-13, IL-15, IL-17, eotaxin, FGF basic, G-CSF, GM-CSF, IFN-γ, IP-10, MCP-1, MIP-1α, PDGF-BB, MIP-1β, RANTES, TNF-α, and VEGF) were chosen and their levels were determined using the Bio-Plex Pro Human Cytokine 27-plex assay. The concentration of the selected cytokines from the cytokine biosignature set were also detected and analyzed using the bio-plex assay, but with a customized human cytokine panel. The bio-Plex assay was performed according to the manufacturer’s instructions. Briefly, the assay was performed at a serum dilution of 1:4 and 50 μL were added to each well. Next, mixed microbeads (50 μL) were added and the 96-well plates were incubated and mixed for 30 min, followed by the incubation with detection antibodies and Streptavidin-PE, with washes after each step. The beads were resuspended in the plate using 125 μL of assay buffer and analyzed using the bio-plex 200 system. The data was obtained and analyzed using bio-plex manager software (version 6.1).

### Data management and statistical analysis

The TB antigen-stimulated cytokine response was defined by subtracting the cytokine concentration in unstimulated serum from the cytokine concentration in ESAT-6 and CFP-10 stimulated serum, as detected by the bio-plex assay. The unstimulated cytokine response was defined as the cytokine concentration detected in unstimulated serum from whole blood assays.

Mann–Whitney tests were used to compare the differences between two groups. The Kruskal–Wallis tests with post-tests (Dunn’s method) were used for testing statistically significant differences in the median values among three groups. Chi squared tests were used to compare the differences in proportions and McNemar’s test was used to compare the difference between paired proportions in the same group. GraphPad Prism 5.01 (GraphPad Software, San Diego, CA) was used for statistical evaluation and graphical representation.

To assess single cytokine performance in diagnosing the different status of TB infections, receiver operating characteristic curves (ROC) were used as described in [20]. The area under the curve (AUC) was calculated and the optimal cut-off values were estimated as the maximum of Youden’s index, defined as sensitivity + specificity − 1. To assess and establish the prediction model for the corresponding disease status based on multiple cytokines, the SVM machine learning algorithm was used within R (R foundation for statistical computing) [21]. The SVM model was built for the first study group (biomarker screening group) and ran in an independent group (biomarker validation group) to prevent over-fitting the predictive signature. In this study, the radial kernel was used to estimate the optimal model parameters [22]. The sensitivity, specificity, and accuracy of the classifier were evaluated at every recursive elimination step. The smallest number of cytokines that results in the highest classification accuracy was chosen as the TB specific cytokine biosignature. All reported statistically significant differences were determined using a two-sided p value of < 0.05.

## Results

### Demographic and clinical characteristics of the study participants

In the current study, a total of 88 and 216 individuals were recruited in the biomarker screening group and the biomarker validation group, respectively. The demographic and clinical characteristics are described in Table 1. There were no significant differences among the groups regarding age, gender, or the proportion of BCG vaccination. In the biomarker screening group, 15 of the ATB patients were determined to be smear- and culture-positive, while 5 were only culture-positive and 8 were only smear-positive. In the biomarker validation group, 16 ATB patients only had a positive culture for *Mtb*, 21 patients only had a positive AFB and 39 were positive for both (Table 1).

**Table 1:** General information for participants

Characteristic	Biomarker screening			Biomarker validation			Clinical validation		
	ATB	LTBI	CON	ATB	LTBI	CON	ATB	LTBI	CON
Number (no.) of participants	28	34	26	76	69	71	91	24	79
Median age (range)	46 (26–55)	43 (15–62)	39 (21–58)	45 (18–72)	41 (19–67)	43 (21–64)	42 (27–69)	40 (23–74)	39 (21–71)
Males, no. (%)	16 (57.1%)	15 (44.1%)	12 (46.2%)	42 (55.3%)	32 (46.4%)	35 (49.3%)	54 (59.3%)	13 (54.2%)	42 (53.2%)
HIV infected, no. (%)	0 (0%)	0 (0%)	0 (0%)	2 (2.6%)	0 (0%)	0 (0%)	1 (1.1%)	0 (0%)	0 (0%)
BCG status									
Vaccinated	17 (60.7%)	25 (73.5%)	23 (88.5%)	56 (73.7%)	55 (79.7%)	59 (83.1%)	58 (63.7%)	17 (70.8%)	54 (68.4%)
Unvaccinated	6 (21.4%)	5 (14.7%)	2 (7.7%)	17 (22.4%)	12 (17.4%)	8 (11.3%)	29 (31.9%)	5 (20.8%)	20 (25.3%)
Unknown	5 (17.9%)	4 (14.3%)	1 (3.8%)	3 (3.9%)	2 (2.9%)	4 (5.6%)	4 (4.4%)	2 (8.3%)	5 (6.3%)
QFT results									
Negative	3 (10.7%)	0 (0%)	26 (100%)	10 (13.2%)	0 (0%)	71 (100%)	17 (18.7%)	0 (0%)	79 (100%)
Positive	25 (89.3%)	34 (100%)	0 (0%)	66 (86.8%)	69 (100%)	0 (0%)	74 (81.3%)	24 (100%)	0 (0%)
Extrapulmonary TB	4 (14.3%)	–	–	30 (39.5%)	–	–	13 (27.7%)	–	–
Microbiologic test									
AFB positive	23 (82.1%)	–	–	60 (78.9%)	–	–	66 (72.5%)	–	–
Culture positive	20 (71.4%)	–	–	55 (72.4%)	–	–	56 (61.5%)	–	–

*ATB* active tuberculosis, *LTBI* latent tuberculosis infection, *CON* TB uninfected subjects, *AFB* acid fast bacilli

To evaluate the clinical application of these biomarkers, we prospectively recruited 194 patients suspected of ATB in a clinical-oriented study group (clinical validation group). According to the pre-established case definitions, 91 of the recruited patients were finally diagnosed as ATB (only 25 patients had a positive culture for *Mtb*, 35 patients had a positive AFB smear, and 31 patients were positive for both).

### Identification of differentially expressed cytokines in response to TB antigens or in the absence of antigen stimulation in the biomarker screening group

We first evaluated the expression levels of 27 cytokines in samples with different TB infection status including active TB, latent TB infection and TB uninfected controls in the biomarker screening group. Both TB-antigen stimulated and unstimulated cytokine responses were analyzed. For the TB-antigen stimulated cytokine response, the expression levels of IFN-γ, IP-10, IL-2, IL-1Ra, MCP-1, and IL-15 were significantly higher in the ATB and LTBI groups compared to those in the CON group (Table 2). Moreover, the TB antigen-stimulated PDGF-BB expression was significantly lower in the LTBI group than those in both the ATB and the control groups (p = 0.011 and p = 0.0165, respectively, Table 2). Except PDGF-BB, the expression of TB antigen-stimulated cytokines showed no significant differences between the ATB and the LTBI groups.

**Table 2:** TB-antigen stimulated and unstimulated levels of differentially expressed cytokines as measured with the bio-plex assay

Marker^a^	Median levels and 25–75% percentile (pg/mL)			p-value		
	ATB (n = 28)	LTBI (n = 34)	CON (n = 26)	ATB vs CON	LTBI vs CON	ATB vs LTBI
TB-antigen stimulated						
IFN-γ	77.6 (29.4 to 134.7)	30.6 (15.5 to 77.5)	− 1.9 (− 20.4 to 18.3)	< 0.0001	< 0.0001	0.1424
IP-10	10,821 (4405 to 18,052)	6221 (4650 to 10,956)	6.5 (− 86.1 to 215.1)	< 0.0001	< 0.0001	0.0994
IL-2	116.8 (31.1 to 270.6)	137.1 (51.7 to 261.4)	− 0.3 (− 4.1 to 4.5)	< 0.0001	< 0.0001	0.6559
IL-1Ra	1342 (331.4 to 2778)	881.6 (453.0 to 2134)	-23.1 (− 186.3 to 242.5)	< 0.0001	< 0.0001	0.2524
MCP-1	9848 (203.2 to 2640)	1610 (− 5.6 to 3526)	− 26.2 (− 300.4 to 159)	< 0.0001	< 0.0001	0.7718
IL-15	2.0 (− 1.5 to 10.4)	3.6 (− 4.0 to 12.7)	− 2.0 (− 10 to 1.2)	0.0018	0.0062	0.5146
PDGF	51.68 (1.330 to 170.0)	− 41.67 (− 192.5 to 47.75)	111.0 (− 35.15 to 303.4)	0.4832	0.011	0.0165
Unstimulated						
VEGF	225.1 (144.3 to 350.2)	127.3 (86.58 to 195.9)	91.89 (50.18 to 130.1)	< 0.0001	0.022	0.003
MCP-1	3460 (1651 to 4262)	1471 (636.4 to 4000)	729.5 (380.9 to 1404)	< 0.0001	0.0105	0.1356
IL-12 (p70)	57.39 (32.79 to 89.25)	34.05 (24.52 to 55.21)	29.19 (15.74 to 35.83)	< 0.0001	0.0354	0.0344
MIP-1b	3164 (1629 to 9066)	3266 (993 to 6318)	1354 (750 to 2240)	0.0005	0.0115	0.4838
Rantes	6575 (3309 to 9687)	3448 (2255 to 7981)	1905 (1608 to 2893)	< 0.0001	0.0002	0.0649
IFN-γ	129.5 (68.27 to 216.6)	95.21 (48.73 to 150.7)	53.32 (31.47 to 110.7)	0.0012	0.0305	0.0298
IP-10	2025 (1460 to 2786)	1138 (473.8 to 2571)	671.2 (446.9 to 1166)	< 0.0001	0.0746	0.0147
IL-9	70.08 (44.87 to 139.1)	51.1 (31.56 to 94.33)	33.66 (21.72 to 59.4)	0.0005	0.0771	0.0937
TNF-α	473 (161.2 to 1007)	218.6 (90.05 to 745)	190.5 (57.65 to 373)	0.0112	0.224	0.091
FGF	54.44 (44.94 to 66.6)	41.76 (26.95 to 73.65)	36.7 (23.25 to 61.72)	0.0267	0.2894	0.0501

*ATB* active tuberculosis, *LTBI* latent tuberculosis infection, *CON* TB uninfected subjects

^a^Only cytokines that were differentially expressed between any two groups of the ATB, LTBI, and CON are listed

**Fig. 2:**
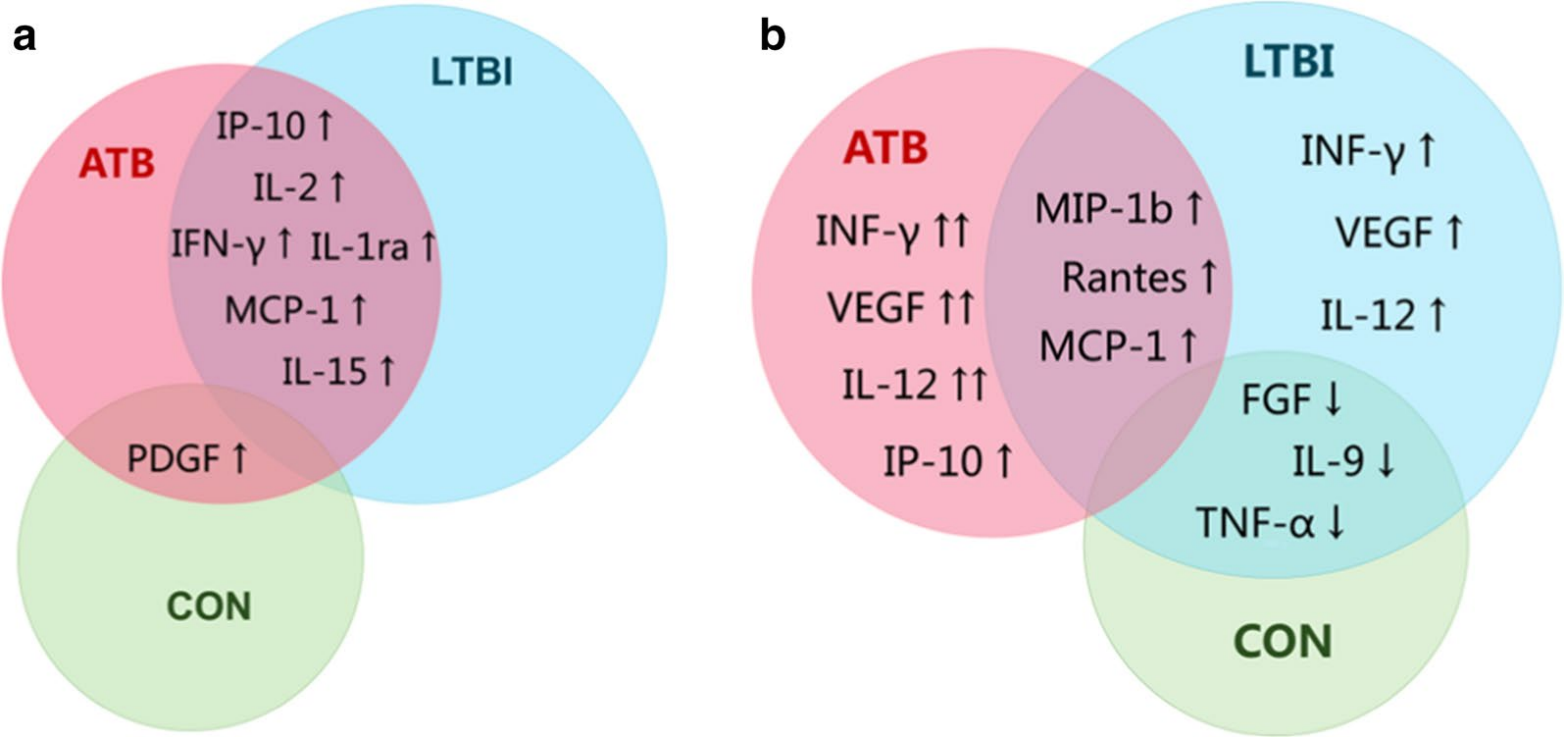
Comparison of TB-antigen stimulated and unstimulated cytokine responses. The comparison was carried out in patients with active TB (ATB), subjects with latent TB infection (LTBI), and TB non-infected controls (CON). The TB-antigen stimulated cytokine response (**a**) was calculated by subtracting the cytokine concentration in unstimulated serum from the cytokine concentration in TB-antigen stimulated serum, as detected by the Bio-Plex assay. The unstimulated cytokine response (**b**) was defined as the cytokine concentration detected in unstimulated serum. The Kruskal–Wallis tests with Dunn’s post-test were used to compare differences among three groups. TB-antigen stimulated (**a**) and unstimulated (**b**) cytokines biomarkers were found to differ significantly between groups. The arrow preceding each biomarker name indicates increased or decreased plasma concentrations in the ATB, LTBI, and CON groups

In unstimulated samples, the expression of IP-10, VEGF, MCP-1, IL-12, MIP-1b, RANTES, and IFN-γ was significantly higher in the ATB and LTBI groups when compared to that in the CON group (Fig. 2). Four cytokines were differentially expressed between the ATB and LTBI groups, including unstimulated VEGF, IL-12, IFN-γ, and IP-10, which were significantly more highly expressed in the ATB group than in the LTBI group (Table 2).

To determine the diagnostic potential of differentially expressed cytokines from previous analyses for detecting infection with *Mtb*, the ROC methodology was used. For this purpose, we combined the data from the ATB and LTBI groups into the TB infected group (TBI; case values) and compared it with the data from the CON group (control values). The TB-antigen stimulated IFN-γ, IP-10, IL-2, MCP-1, IL-1Ra, and IL-15 levels and the unstimulated VEGF, IP-10, IL-12 (p70), IFN-γ, MCP-1, and MIP-1b levels were found to be significantly higher in the TBI group than in the CON group (Additional file 1). The diagnostic performance (i.e., the AUC, cut-off value, sensitivity, and specificity) of these cytokines based on ROC analyses is shown in Additional file 2. Among all cytokines, the TB-antigen stimulated IP-10 levels represented the strongest discriminator between the TBI and CON groups, with an AUC of 0.9945 (sensitivity: 90.3%, specificity: 100.0%). Furthermore, the TB-antigen stimulated IL-2, MCP-1, and IL-1Ra also achieved sensitivity and specificity values close to or exceeding those of IFN-γ (Table 4 and Additional file 2).

Since current diagnostic methods (QFT and T-SPOT.*TB*) can be used to reliably identify TB-negative individuals, we next decided to remove the CON group and focus on assessing biomarkers for discriminating between the ATB and LTBI groups. The differentially expressed cytokines between the ATB and LTBI groups were then selected and ROC analyses were performed to evaluate their diagnostic potential. The results are shown in Additional file 2 (ATB as the case value and LTBI as the control value). The unstimulated VEGF exhibited the highest AUC (0.8106) and correctly classified 53.6 and 91.2% of the participants into the ATB and LTBI groups, respectively. The unstimulated IP-10, IL-12, and IFN-γ displayed relatively high AUCs for discriminating between the ATB and LTBI groups (0.7717, 0.7476, and 0.7276, respectively).

### Development and validation of the cytokine signature for discriminating between different stages of TB infection

According to our results from the biomarker screening group, no single cytokine achieved a good enough diagnostic performance for it to be considered as a diagnostic biomarker. In order to identify a subset of differentially expressed cytokines that could discriminate between the different stages of the *Mtb* infection, we first subjected our cytokine expression data from the biomarker screening group to SVM analysis. This analysis determined that the most parsimonious model required only six of these cytokine biomarkers to accurately assign 97.7% (86/88) of the subjects to their respective groups (Table 3). These cytokines included the TB-antigen stimulated IFN-γ, IP-10, IL-1Ra (IFN-γA, IP-10A, IL-1RaA) levels and the unstimulated IP-10, VEGF, IL-12 (p70) (IP-10N, VEGFN, IL-12N) levels. Using the six-cytokine expression biosignature, the sensitivity was 96.4% (27/28) and specificity was 98.3% (59/60) for discriminating the ATB group from the LTBI and CON groups (Table 3). The AUC for the six-cytokine biosignature (determined from the biomarker screening group) was 0.9817 (95% CI 0.9623–1.001).

**Table 3:** Accuracy of the six-cytokine biosignature in the diagnosis of active TB

	Sensitivity	Specificity	PPV	NPV	Accuracy
Biomarker screening group (n = 88)					
%, (n/N)	96.4 (27/28)	98.3 (59/60)	96.4 (27/28)	98.3 (59/60)	97.7 (86/88)
95% CI	80.8–100.0	90.3–100.0	80.8–100.0	90.3–100.0	91.6–99.9
Biomarker validation group (n = 216)					
%, (n/N)	88.2 (67/76)	92.1 (129/140)	85.9 (67/78)	93.5 (129/138)	90.7 (196/216)
95% CI	78.8–93.9	86.3–96.7	76.3–92.1	87.9–96.7	86.1–94.0
Clinical validation group (n = 194)					
%, (n/N)	85.7 (78/91)	91.3 (94/103)	89.7 (78/87)	87.9 (94/107)	88.7 (172/194)
95% CI	76.9–91.6	84.0–95.5	81.3–94.7	80.2–92.9	83.4–92.5

*NPV* negative predictive value, *PPV* positive predictive value

**Fig. 3:**
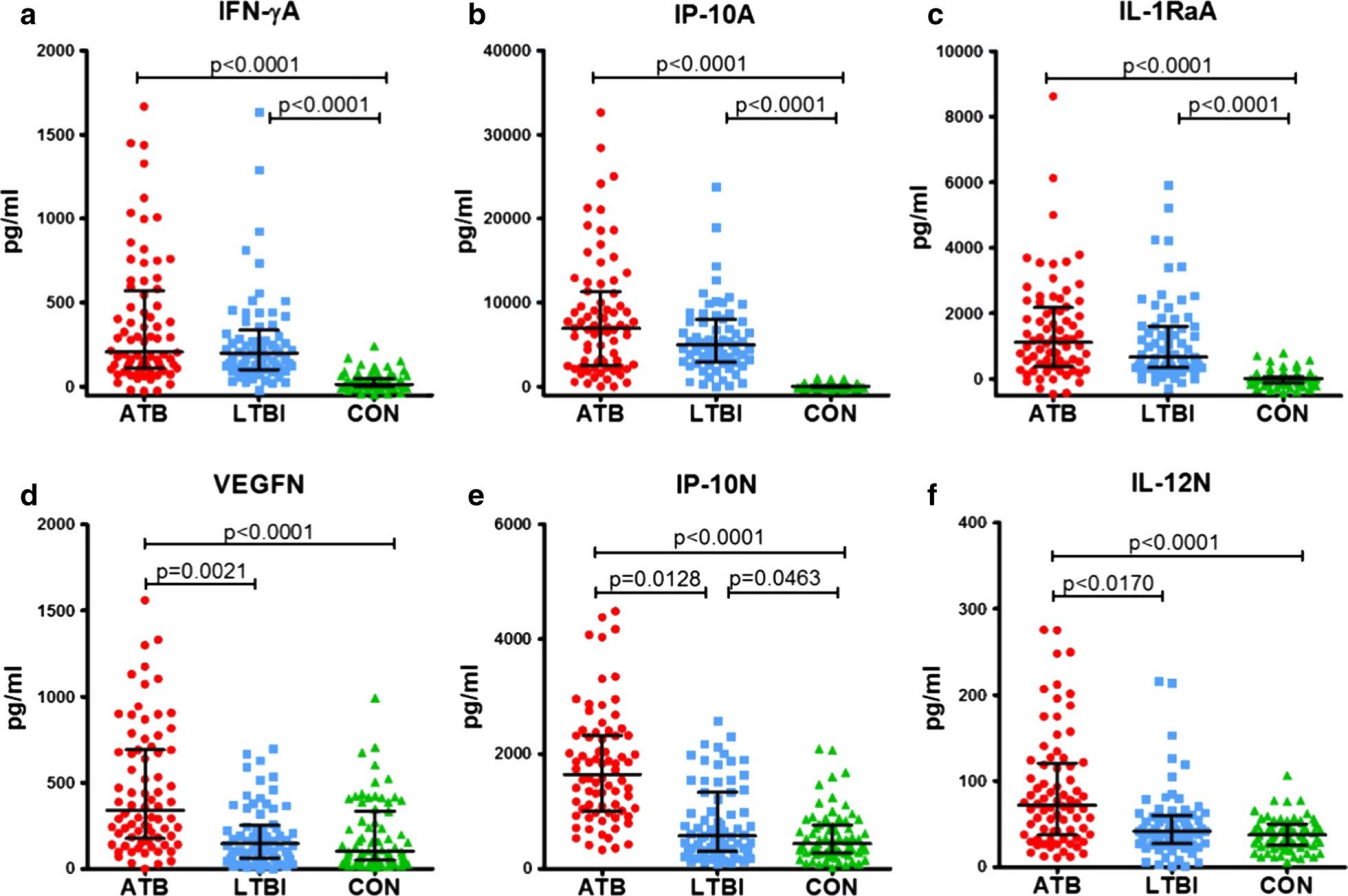
Cytokine levels detected from serum samples in the biomarker validation group. Levels of cytokines detected in serum samples from patients with active TB (ATB, n = 76), individuals with latent TB infection (LTBI, n = 69), and TB non-infected controls (CON, n = 71) in the biomarker validation group. Representative plots for TB antigen stimulated IFN-γ (IFN-γA), IP-10 (IP-10A), IL-1Ra (IL-1RaA) (**a**–**c**) and unstimulated VEGF (VEGFN), IP-10 (IP-10N), IL-12 (p70) (IL-12N) (**d**–**f**) are shown. Error bars in the scatter-dot plots indicate the medians and IQRs

To better assess the diagnostic performance of the six-cytokine biosignature, we then recruited an independent cohort (biomarker validation group) including 76 patients with ATB, 69 LTBI, and 71 CON subjects. We assessed the expression levels of discriminant cytokines using the bio-plex assay and a customized panel including these cytokines. We found that the expression pattern of the six-cytokine biosignature was similar to our previous results in the biomarker screening group (Fig. 3 and Additional file 3). As shown in Fig. 3, expression of IFN-γA, IP-10A and IL-1RaA were significantly higher in ATB and LTBI group than CON group, but showed no significant difference between ATB and LTBI. For IP-10N, VEGFN and IL-12N, the expression was significantly higher in ATB group than both LTBI and CON group, while significant differences were also observed between ATB and LTBI. When integrating cytokine expression levels, the discriminant six cytokine biosignature assigned 196 of the 216 (90.7%) subjects to the correct groups in a blinded analysis (Table 3). When combining the data from the LTBI and CON groups in a control group, this six-cytokine biosignature ascertained the presence of ATB with a sensitivity of 88.2% (67/76) and specificity of 92.1% (129/140) in the biomarker validation cohort (Table 3). The AUC of the six-cytokine biosignature was 0.9126 (95% CI 0.8874–0.9423) for identifying ATB patients.

### Clinical application of the six-cytokine biosignature in cohorts of TB suspects

To examine the diagnostic performance of the six-cytokine biosignature for clinical applications, a prospective study was conducted on 194 patients with suspected ATB recruited at Huashan hospital, Shanghai. At enrollment, all 194 patients were tested using the AFB smear, QFT, and the six-cytokine biosignature test. Among the 194 enrolled subjects, 91 were finally diagnosed as ATB if they met one of the ATB-indicative criteria (see “Methods” for details).

**Fig. 4:**
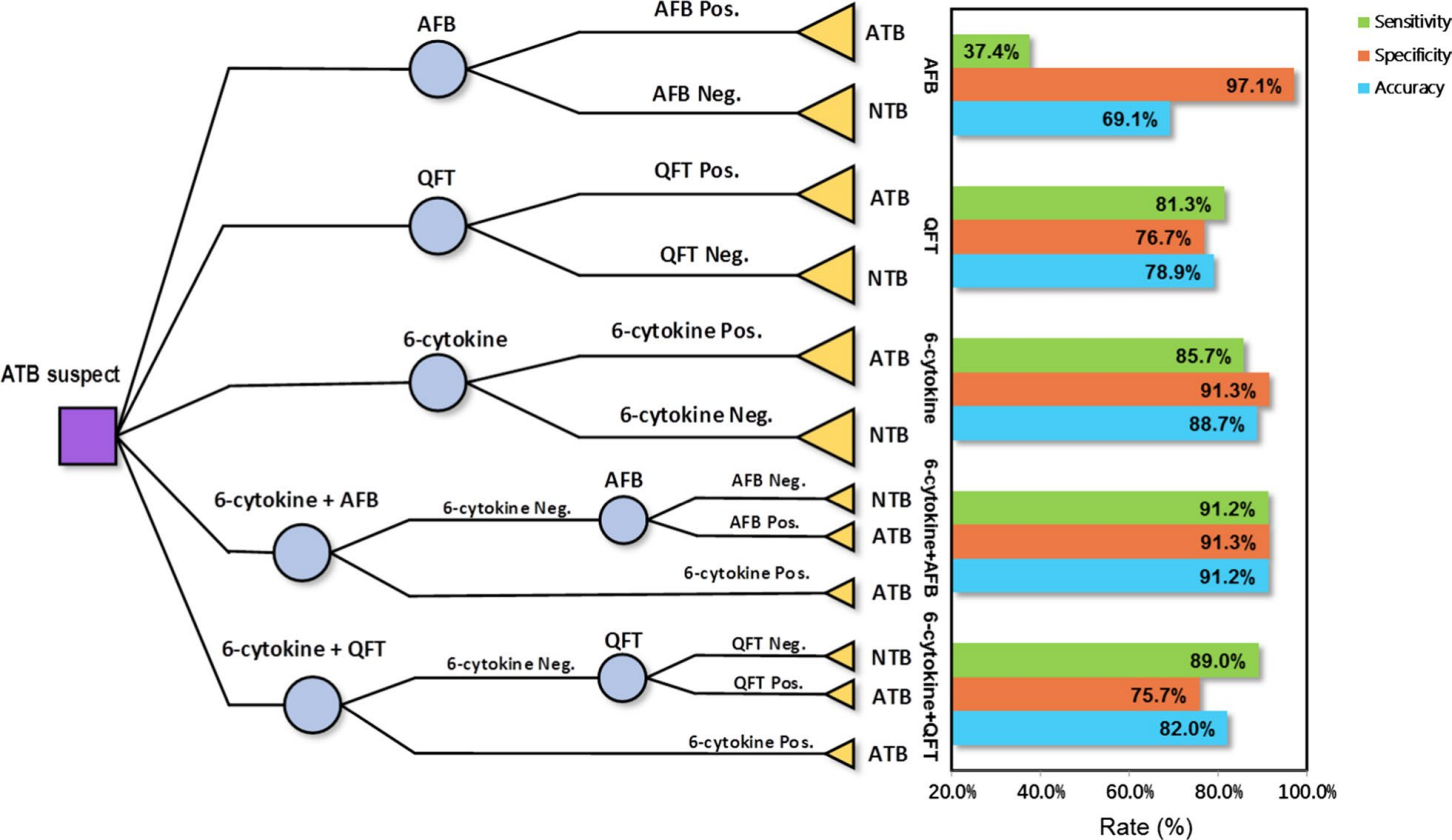
Study decision tree and diagnostic performance of single and combined tuberculosis (TB) diagnostic tests. The left side depicts the decision analysis of different diagnostic tests in the clinical validation cohort. The square represents a decision node, circles represent chance nodes, and triangles indicate terminal nodes. The right side of this graph shows the sensitivity, specificity, and accuracy of the diagnostic performance of each diagnostic test alone, and the additional gain when combining the six-cytokine biosignature test with either AFB or QFT in ATB suspects from a clinical validation cohort. *AFB* acid fast bacilli, *QFT* QuantiFERON TB GOLD in-tube, *6-cytokine* six-cytokine biosignature test, *Pos.* positive; *Neg.* negative, *ATB* patients with active TB, *NTB* subjects without ATB disease

We then evaluated the diagnostic accuracy of the six-cytokine biosignature in classifying all study participants into the ATB or “NTB” groups (including latently infected or TB uninfected individuals), but blinded for the reference standard. In addition, as shown in the left side of Fig. 4, we compared this approach with other diagnostic methods, including the AFB smear and QFT. The six-cytokine biosignature test was also combined with AFB or QFT in a two-step diagnostic procedure: a positive result was determined when either of the test results was positive, and a negative result was assigned when both test results were negative (Fig. 4).

The sensitivity, specificity and diagnostic accuracy of the six-cytokine biosignature test, QFT, AFB and two combined tests were shown in the right part of Fig. 4. Our results revealed that our six-cytokine biosignature test displayed superior performance in discriminating ATB from NTB individuals with a sensitivity and specificity of 85.7% (78/91) and 91.3% (94/103), respectively (Table 3 and Fig. 4). When compared with other tests, the six-cytokine biosignature test demonstrated a significantly higher sensitivity than AFB (McNemar’s test, p < 0.0001) and a higher specificity than QFT (McNemar’s test, p < 0.0001) (Fig. 4). The combination of the six-cytokine biosignature test and AFB led to a combined sensitivity of 91.2% (83/91) and specificity of 91.3% (94/103), with a diagnostic accuracy of 91.2% (177/194). Thus, the sensitivity was higher than the six-cytokine biosignature test alone, but the difference was not significant. However, a combination of the six-cytokine test and QFT achieved a relatively high sensitivity (89.0%, 81/91), the specificity was still not improved (75.7%, 78/103).

## Discussion

In this study, we examined the potential of 27 cytokines to diagnose and differentiate between the various stages of the TB infection and TB non-infected controls. One of the most fundamental aspects of establishing an immunodiagnostic test is the selection of stimuli or antigens. Initially, *Mtb*-derived purified protein derivatives (PPD) have been used in IGRAs. As a mixture of *Mtb* antigens, PPDs could stimulate more diverse TB-specific immune responses than single antigens [19]. However, PBMCs from BCG-vaccinated subjects may exhibit a strong cross-reaction when treated with PPD, which will lead to a false positive result. Therefore, the RD1 encoded ESAT-6 and CFP-10, being absent from BCG strains and most non tuberculous mycobacteria, were chosen as antigens in our cytokine release assays to improve the diagnostic specificity [6, 23].

In addition to the TB-antigen stimulated cytokine levels, unstimulated cytokines such as IP-10, VEGF, and IL-12 from peripheral blood samples showed a promising diagnostic potential for distinguishing between ATB and LTBI (Table 2). Moreover, without antigen stimulation in vitro, the cytokine detection assays could be easily and rapidly performed, which might be more beneficial than antigen stimulation assays for the management of TB in resource-limited areas. It is worth noting that the peripheral circulating cytokine profiles may be modulated by multiple factors aside from the TB infection [12, 24], such as other bacterial or viral infections. The TB-antigen stimulated cytokine profiles represent *Mtb*-specific cytokine expressions, which can accurately detect TB-infected individuals (including both ATB and LTBI) first without being affected by other factors. Therefore, the unstimulated cytokine profiles should be assessed in combination with TB-antigen stimulated cytokines to avoid aberrant results. Another advantage of using multiple biomarkers in combination is to derive a unified assay that could reduce the impact of the plasticity of the immune system and the heterogeneity of cytokine responses to infections [12]. This could improve the diagnostic accuracy for patients with diverse immune backgrounds, especially in clinical settings.

The multiplexed assays in our study demonstrated that in addition to IFN-γ, four cytokines (IL-2, MCP-1, IL-1Ra, and IP-10) were identified as potential alternative biomarkers for detecting the *Mtb* infection. Among these cytokines, the diagnostic potential of IL-2 has been evaluated by several studies as a biomarker for the diagnosis of both active and latent TB infection. A meta-analysis of these studies showed that the IL-2 release assay can improve the diagnostic ability of IGRAs to identify individuals with ATB and LTBI [25]. Consistent with the findings of these studies, we also found that TB-antigen stimulated IL-2 releases were significantly higher in TB-infected individuals (ATB + LTBI) than individuals in CON group. These results all indicate IL-2 as a promising biomarker for detecting TB infection. MCP-1, also known as CCL2, belongs to the CC chemokine family, which could recruit monocytes, memory T cells, and dendritic cells to the sites of inflammation after bacterial infection. Several case–control studies suggest that the expression of MCP-1 in response to specific *Mtb* antigen stimulation was significantly higher in patients with ATB than in healthy controls [26–28]. However, these studies also indicated that the antigen-stimulated MCP-1 expression is heterogeneous in patients with ATB, which is consistent with our results. Thus, it is necessary to employ MCP-1 in combination with other cytokines to improve diagnostic performance.

Although the TB-antigen stimulated IL-2, MCP-1, IP-10 and IL-1Ra all showed high diagnostic performance for detecting TB infection in the biomarker screening group, only IP-10 and IL-1Ra were included in the six-cytokine biosignature by SVM analysis. Since SVM were used for the selection of biomarkers that resulted in the highest classification accuracy, the selection indicated that TB antigen stimulated IP-10 and IL-1Ra contributed more to the predictive value of the multi-cytokine signature than IL-2 and MCP-1 [21, 29]. IL-1Ra is a naturally occurring competitive inhibitor of IL-1a and IL-1b. Serum IL-1Ra has been proposed as a biomarker for TB infection which declines with treatment [30]. Similar to our results, the differential expression of TB-antigen stimulated IL-1Ra between TB-infected individuals and healthy controls has also been reported in other studies [28, 31]. In addition to our research, most of these studies concerning the diagnostic performance of IL-1Ra are preliminary. Future studies are still needed to assess the diagnostic value of IL-1Ra in large-scale and multi-site prospective cohort.

IP-10, which is a chemokine secreted by antigen presenting cells upon stimulation by multiple cytokines including IFN-γ and TNF-α, has been extensively investigated as an alternative biomarker for TB infection [32, 33]. The release of IP-10 after stimulation by ESAT-6 and CFP-10 has been shown to have comparable sensitivity and specificity with IGRAs and could be combined with IFN-γ to improve diagnostic accuracy. Furthermore, as an alternative diagnostic biomarker, IP-10 was expressed at higher levels than IFN-γ, which may be a more robust marker in young children and in HIV-infected individuals with low CD4 cell counts [32]. However, the *Mtb*-specific release of IP-10 cannot be used to distinguish between ATB and LTBI [20, 32]. In the present study, both the TB-antigen stimulated and unstimulated IP-10 expression, were determined as candidate biomarkers for our six-cytokine biosignature. As one of the key cytokines in human immune responses to *Mtb* infection, it is not surprising that more IP-10 could be detected in the peripheral blood of patients with ATB [32, 34]. Compared with the typical readout biomarkers such as IFN-γ, antigen stimulated IP-10 is expressed in much higher levels. This important distinction may also enhance its ability to amplify the differences in host immune responses between actively and latently infected subjects [32]. It is worth nothing that the serum (unstimulated) IP-10 level was significantly higher in the ATB group than in both the LTBI and CON groups. The differential expression of serum IP-10 was also found in several studies [32, 34–37]. In our study, it was further confirmed that serum IP-10 was a potential diagnostic biomarker in distinguishing between ATB and LTBI.

In our study, serum VEGF, IL-12, and IP-10 were found to be differently expressed between the ATB and LTBI groups, which made them potential biomarkers for discriminating between these two groups. VEGF, originally known as vascular permeability factor, is a multifunctional cytokine that is involved in angiogenesis and vascular permeability [38]. Several studies have reported that VEGF is substantially overexpressed in patients with ATB and serum VEGF levels were significantly higher in patients with active pulmonary tuberculosis than in patients with old tuberculosis or no-TB controls [39–41]. Similar results were also observed in extrapulmonary tuberculosis such as tuberculous pleurisy [42] or tuberculous meningitis [43]. The increased VEGF levels in the serum may possibly be due to the increased production of VEGF by macrophages, epithelioid cells, and other inflammatory cells around active tuberculosis lesions to supplement the blood supply [39, 40]. In this study, we also observed higher VEGF levels in patients with ATB, which were nearly two fold those in TB non-infected controls as well as subjects with LTBI. Our results indicate that VEGF may be a useful screening marker for discriminating ATB from LTBI. However, the overexpression of VEGF can be also found in a high percentage of malignant animal and human tumors [44], such as lung cancer, which may lead to a relatively low specificity [40] and affect the diagnostic accuracy. In combination with other cytokines stimulated by *Mtb*, specific antigens may help to address such problems. In addition, since VEGF levels decrease with the duration of the therapy [39–41], further studies involving a larger number of patients with tuberculosis are needed to evaluate the diagnostic performance of VEGF in assessing treatment response.

As a Th1-type cytokine, IL-12 might play a crucial role in regulating IFN-γ production and the cytotoxic effector function of *Mtb* antigen-specific T cells [45]. The IL-12 response during anti-TB treatment has been investigated by several studies, which found a reduction in IL-12 levels over the treatment course [46, 47]. In our study, increased IL-12 levels were found in the serum of patients with ATB. This may suggest a key role for IL-12 in the development of cell-mediated immunity [48], which was increased after *Mtb* infection. However, the diagnostic potential of IL-12 needs to be further investigated.

This study has several limitations. First, the relatively small sample size may lead to wide uncertainty intervals and to overestimating the diagnostic accuracy of the selected biomarkers. Multi-site, longitudinal cohort studies with a larger sample size and broader range of disease are still required. Furthermore, a larger number of subjects, including those with immunodeficient conditions such as children, HIV-infected subjects and patients undergoing immunosuppressive therapy should be recruited in future studies. The predictive value of any cytokine biomarker for the progression from LTBI to ATB remains uncertain and requires further evaluation. Additionally, for the clinical introduction of these new biomarkers in places with poor medical infrastructure, simpler and less costly detection methods such as ELISA need to be developed and evaluated. In addition to IFN-γA, IP-10A, IL-1RaA and IP-10N, VEGFN, IL-12N, a number of other cytokines had also been found with high diagnostic potential in the biomarker screening group, including TB-antigen stimulated IL-2, MCP-1, PDGF-BB and unstimulated IFN-γ. Although they were not further validated in our study because they were not selected into the multi-cytokine biosignature by SVM analysis, their diagnostic values are still worthy of further study.

## Conclusions

Our study identified a number of *Mtb*-specific cytokine responses associated with different stages of TB infection. A six-cytokine biosignature selected from these cytokines had promising diagnostic performance at identifying TB infected individuals (including both ATB and LTBI). Importantly, the six-cytokine biosignature could also distinguish between ATB and LTBI with high levels of accuracy. Moreover, our cytokine combination has been validated in a clinically based prospective cohort. We constantly endeavor to optimize diagnostic tools for TB, as the identification of active cases and subsequent treatment represent the central pillar of the End TB Strategy.

## Additional files

**Additional file 1.** ROC analysis of the differentially expressed cytokines to discriminate between TB infected individuals (active TB patients and latent infected subjects) and TB uninfected controls.

**Additional file 2.** ROC analysis of the differentially expressed cytokines to discriminate between ATB and LTBI.

**Additional file 3.** Different expressed levels of the 6-cytokine signature in ATB, LTBI and CON group from the biomarker validation cohort.

## Abbreviations

TB: tuberculosis
*Mtb*: *Mycobacterium tuberculosis*
LTBI: latent tuberculosis infections
ATB: active tuberculosis
IFN-γ: interferon-γ
IGRAs: interferon-γ release assays
ELISA: enzyme-linked immunosorbent assay
QFT: QuantiFERON-Gold in tube
PBMC: peripheral blood mononuclear cell
RD: region of difference
ESAT-6: early secretory antigenic target
CFP: culture filtrate protein
BCG: Bacillus Calmette–Guérin
SVM: support vectors machine
AFB: acid fast bacilli
NTB: no active tuberculosis
ROC: receiver operating characteristic curves
AUC: area under the curve
PPD: purified protein derivatives
