# Supplementary material for: Screening and identification of a six-cytokine biosignature for detecting TB infection and discriminating active from latent TB
Source: J Transl Med. 2018 Jul 20;16:206. doi: 10.1186/s12967-018-1572-x (PMC6054748; doi:10.1186/s12967-018-1572-x)
Supplement: Supplementary file 1 — Additional file 1. ROC analysis of the differentially expressed cytokines to discriminate between TB infected individuals (active TB patients and latent infected subjects) and TB uninfected controls. [file 12967_2018_1572_MOESM1_ESM.docx]

**Additional file 1.** ROC analysis of the differentially expressed cytokines to discriminate between TB infected individuals (active TB patients and latent infected subjects) and TB uninfected controls.

| Marker | Cut-off(pg/ml) | AUC | Sensitivity % | | | Specificity% |
| --- | --- | --- | --- | --- | --- | --- |
|  |  |  | ATB | LTBI | TBI(ATB+LTBI) | CON(n=26) |
|  |  |  | (n=28) | (n=34) | (n=62) |  |
| TB-antigen stimulated | |  |  |  |  |  |
| IFN-γ | 31.5 | 0.8214 | 75 | 47.1 | 59.7 | 88.5 |
| IP-10 | 2009 | 0.9945 | 92.9 | 88.2 | 90.3 | 100 |
| IL-2 | 14.3 | 0.9794 | 92.9 | 85.3 | 88.7 | 96.2 |
| MCP-1 | 197.5 | 0.8228 | 78.6 | 70.6 | 74.2 | 80.8 |
| IL-1ra | 641 | 0.8516 | 71.4 | 55.9 | 62.9 | 92.3 |
| IL-15 | 6 | 0.7486 | 35.7 | 41.2 | 38.7 | 92.3 |
| Unstimulated |  |  |  |  |  |  |
| VEGF | 150.5 | 0.8929 | 75 | 47.1 | 59.7 | 88.5 |
| IP-10 | 1384 | 0.8736 | 78.6 | 41.2 | 58.1 | 84.6 |
| IL-12(p70) | 43.9 | 0.8283 | 64.3 | 38.2 | 50 | 92.3 |
| IFN-γ | 130.4 | 0.7576 | 50 | 29.4 | 38.7 | 88.5 |
| MCP-1 | 2526 | 0.8462 | 64.3 | 44.1 | 53.2 | 96.2 |
| MIP-1b | 3305 | 0.7761 | 50 | 50 | 50 | 92.3 |
