# Supplementary material for: Screening and identification of a six-cytokine biosignature for detecting TB infection and discriminating active from latent TB
Source: J Transl Med. 2018 Jul 20;16:206. doi: 10.1186/s12967-018-1572-x (PMC6054748; doi:10.1186/s12967-018-1572-x)
Supplement: Supplementary file 2 — Additional file 2. ROC analysis of the differentially expressed cytokines to discriminate between ATB and LTBI. [file 12967_2018_1572_MOESM2_ESM.docx]

**Additional file 2**. ROC analysis of the differentially expressed cytokines to discriminate between ATB and LTBI.

| Marker | AUC | Cut-off(pg/ml) | Sensitivity % | Specificity % |
| --- | --- | --- | --- | --- |
|  |  |  | ATB(n=28) | LTBI(n=34) |
| TB-antigen stimulated |  |  |  |  |
| PDGF | 0.7686 | 2.23 | 60.7(17/28) | 85.3(29/34) |
| Unstimulated |  |  |  |  |
| VEGF | 0.8106 | 115.9 | 53.6(15/28) | 91.2(31/34) |
| IP-10 | 0.7717 | 956.1 | 46.4(13/28) | 91.2(31/34) |
| IL-12 | 0.7476 | 54.26 | 67.9(15/28) | 73.5(25/34) |
| IFN-γ | 0.7276 | 118.3 | 64.3(18/28) | 76.5(26/34) |
