# Supplementary material for: Screening and identification of a six-cytokine biosignature for detecting TB infection and discriminating active from latent TB
Source: J Transl Med. 2018 Jul 20;16:206. doi: 10.1186/s12967-018-1572-x (PMC6054748; doi:10.1186/s12967-018-1572-x)
Supplement: Supplementary file 3 — Additional file 3. Different expressed levels of the 6-cytokine signature in ATB, LTBI and CON group from the biomarker validation cohort. [file 12967_2018_1572_MOESM3_ESM.docx]

**Additional file 3.** Different expressed levels of the 6-cytokine signature in ATB, LTBI and CON group from the biomarker validation cohort.

| **Marker** | **Median levels and 25%-75% percentile** | | | **P-value** | | |
| --- | --- | --- | --- | --- | --- | --- |
|  | **ATB (n=76)** | **LTBI (n=69)** | **CON (n=71)** | **ATB vs CON** | **LTBI vs CON** | **ATB vs LTBI** |
| **IFN-g A** | 207.2(109.6-570.6) | 198.5(101.5-335.4) | 14.68(3.2-47.3) | <0.0001 | <0.0001 | 0.1894 |
| **IP-10 A** | 6941(2502-11282) | 4958(2924-8006) | 12.01(-37.57-165.2) | <0.0001 | <0.0001 | 0.109 |
| **IL-1ra A** | 1120(383.3-2175) | 665.6(354.6-1610) | 11.4(-118.6-86.34) | <0.0001 | <0.0001 | 0.1781 |
| **VEGF N** | 338.4(175.6-690.4) | 145.2(62.01-252.6) | 101.8(48.37-335.1) | <0.0001 | 0.6317 | 0.0021 |
| **IP-10 N** | 1645(1013-2318) | 571.4(301.1-1335) | 436.8(270.8-761.3) | <0.0001 | 0.0463 | 0.0128 |
| **IL-12 N** | 71.64(37.39-120.7) | 41.6(26.87-59.67) | 37.28(25.22-50.29) | <0.0001 | 0.139 | 0.0170 |
